# Adult Learning and Employment Opportunities for Older Workers in Australia and the United States: Lessons for Adult Education

**DOI:** 10.1093/geroni/igab046.561

**Published:** 2021-12-17

**Authors:** Phyllis Cummins, Philip Taylor, Takashi Yamashita, Leah Janssen

**Affiliations:** 1 Miami University, Oxford, Ohio, United States; 2 Federation University Australia, Berwick, Victoria, Australia; 3 University of Maryland, Baltimore County, Baltimore, Maryland, United States; 4 Scripps Gerontology Center, Oxford, Ohio, United States

## Abstract

This study examined the role community colleges (U.S.) and Technical and Further Education (TAFE; Australia) institutes play in providing educational opportunities to older workers in the U.S. and Australia. Employment for adults of all ages has been impacted by job automation in recent decades. We analyzed national level data to estimate the impacts of job automation by age group. In both the U.S. and Australia, about 65% of older workers in sales occupations are at risk for job loss due to automation. Additionally, we reviewed occupational projection data and employment opportunities for workers who may be displaced by automation. Needs for health care support occupations, such as nursing assistants and occupational and physical therapy assistants are expected to grow rapidly. We will provide several recommendations based on the integration of our findings related to education/training programs and the aging workforce in the context of community colleges and TAFEs.

